# Lack of association between the *TMPRSS6* gene polymorphism (rs855791) and anemia: a comprehensive meta-analysis

**DOI:** 10.1016/j.htct.2025.103737

**Published:** 2025-03-12

**Authors:** Jethendra Kumar Muruganantham, Ramakrishnan Veerabathiran

**Affiliations:** Human Cytogenetics and Genomics Laboratory, Faculty of Allied Health Sciences, Chettinad Hospital and Research Institute, Chettinad Academy of Research and Education**,** Kelambakkam, Tamilnadu 603103, India

**Keywords:** Genetics, Anemia, Polymorphisms, *TMPRSS6 gene*, Iron deficiency

## Abstract

**Background:**

Anemia affects around 1.6 billion people worldwide and presents a significant challenge for healthcare providers. Despite the hemoglobin concentration being commonly used for diagnosis, identifying underlying causes remains challenging, particularly in vulnerable groups like children under five and pregnant women. Genetic factors, notably variations in the *TMPRSS6* gene, are implicated in iron deficiency anemia, yet the precise relationship with anemia remains unclear.

**Methods:**

A thorough literature search was conducted across databases, including Embase, Google Scholar, and PubMed, focusing on studies investigating *TMPRSS6* gene polymorphisms and anemia. Thirteen eligible studies, comprising 2082 cases and 2684 controls, underwent meta-analysis using Review Manager 5.4 software. Various genetic models were assessed, including allelic, homozygous, heterozygous, dominant, and recessive, with no significant relationship found between the *TMPRSS6* rs855791 polymorphism and anemia.

**Conclusion:**

This meta-analysis provides robust evidence suggesting no significant association between the *TMPRSS6* rs855791gene polymorphism and anemia. These findings underscore the complexity of genetic factors contributing to anemia and emphasize the importance of the further investigation to unravel the mechanisms underlying this relationship for improved diagnostic and therapeutic approaches.

## Introduction

About 1.6 billion individuals worldwide are affected by anemia, a common hematological disorder encountered by general practitioners and hospital doctors.^1^ Using the patient's hemoglobin (Hb) concentration to diagnose anemia is common practice, yet this information alone may not pinpoint the underlying disease responsible for the anemia.^2^ Children under five years old and pregnant and non-pregnant women aged 15–49 are considered the most vulnerable groups, with global prevalence estimates indicating rates of 47 %, 42 %, and 30 % for anemia, respectively.^3^ Anemia is not a singular disease but a broad spectrum of pathological disorders. It is defined functionally and quantitatively as a state where there are insufficient erythrocytes (oxygen-carrying blood cells) in the bloodstream to meet metabolic demands. In clinical practice, the identification of anemia relies on measures such as Hb levels, hematocrit, or red blood cell count that falls below the expected norms adjusted for age and sex.^4^ Several factors influence Hb levels, including smoking, residing at high altitudes, and dietary habits. Children in high-altitude regions can refer to normalized Hb value curves for accurate assessment. Moreover, individual genetic differences, as revealed by genome-wide association studies, contribute to variations in erythrocyte indices.^5^ Iron deficiency or anemia in young children can lead to poor growth and failure to thrive, affecting their neurocognitive and behavioral development.^6^ Historically, iron deficiency anemia (IDA) has been associated with environmental factors such as illness and nutrition. Recent studies suggest that genetic factors play a significant role in the development of IDA. Approximately 20–30 % of the variations in iron concentration may be due to genetic factors.^7^ Common single nucleotide polymorphisms (SNPs) that have been replicated and shown to influence inter-individual variance in blood Hb levels are associated with various biological processes. These processes include Hb production, erythropoiesis (the production of red blood cells), and iron metabolism.^8^ Genetic alterations in the gene coding for transferrin (*TF*), transmembrane serine protease 6 (*TMPRSS6*), and solute carrier family 40 member 1 (*SLC40A1*) are the primary sources of genetic diversity leading to iron deficiency. Among these genes, mutations in the *TMPRSS6* gene are frequently linked to reduced iron levels and hematological parameters, such as erythrocyte volumes and Hb concentrations.^9^

The *TMPRSS6* gene, predominantly expressed in the liver, plays a crucial role in regulating iron homeostasis by negatively modulating the synthesis of hepcidin, the master hormone governing iron levels in the body.^10^ Matriptase-2 (MT-2) hinders the expression of hepcidin and is encoded by the *TMPRSS6* gene.^11^ The MT-2 protein domain borders align precisely with the intron/exon junctions across all species in this gene located on chromosome 22, consisting of 18 exons and 17 intervening introns.^12^ Several *TMPRSS6* SNPs were implicated as indicators of low blood indices, such as rs855791 and rs4820268.^13^ The current meta-analysis seeks to elucidate the relationship between the *TMPRSS6* rs855791 variant and the occurrence of IDA.

## Methodology

### Literature search

PubMed, Google Scholar, and Embase were used to search for anemia-related articles with *TMPRSS6* gene, polymorphism, SNPs, and genetic variations as search criteria. The meta-analysis assessed relevant references with predetermined inclusion/exclusion criteria.

### Inclusion and exclusion criteria

As a prerequisite to conducting an accurate meta-analysis, specific criteria were established to determine the suitability of relevant studies. The studies needed a case-control or similar design, focusing on the correlation between the *TMPRSS6* gene and anemia. The studies also had to deliver genotype and allelic frequency data, consistent 95 % confidence intervals (95 % CIs), and p-values for assessing the odds ratio (OR). The Newcastle-Ottawa Scale (NOS) was used to interpret the meta-analysis results. Any research that failed to meet these criteria or had insufficient data was disregarded.

### Data extraction

**Figure 1:**
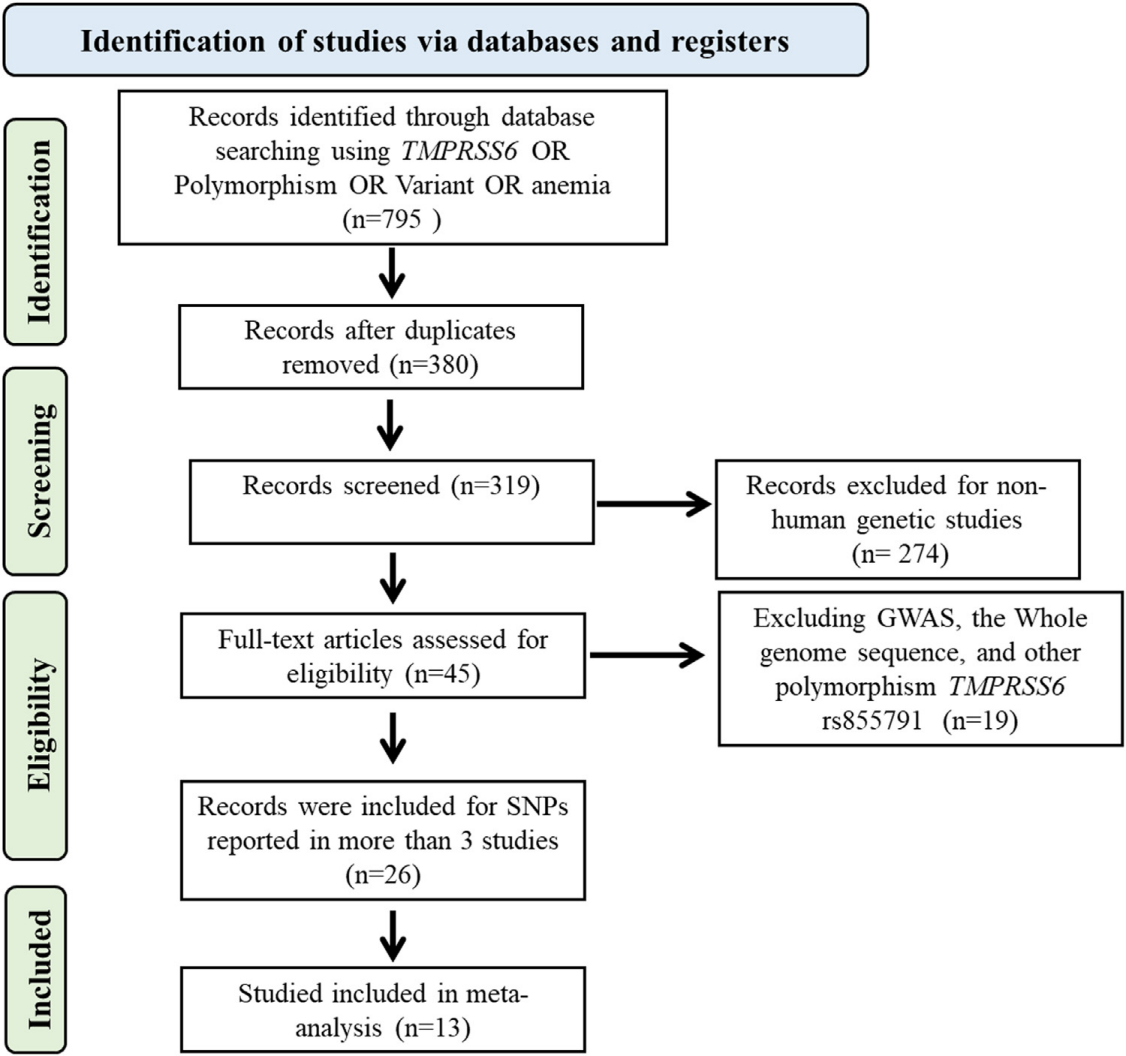
Study selection of the *TMPRSS6* rs855791 gene polymorphism and anemia.

Using the criteria, the researchers systematically gathered relevant papers and conducted data extraction in a standardized manner. A comprehensive examination of available publications was performed to retrieve details on allelic frequencies and genotypes for both case and control participants. In cases where genotypic data was insufficiently provided, it was derived from existing data, such as allelic frequencies. Studies that could not obtain meaningful data from both case and control groups were excluded. The extracted data from each study encompasses the Pubmed ID, study design, publication year, first author name, sample size, ethnicity, Hardy-Weinberg equilibrium (HWE) score, language, and other pertinent information. Figure 1 depicts a Preferred Reporting Items for Systematic Reviews and Meta-Analyses (PRISMA) flowchart that describes how papers were selected and screened for meta-analysis.

### Risk bias

**Figure 2:**
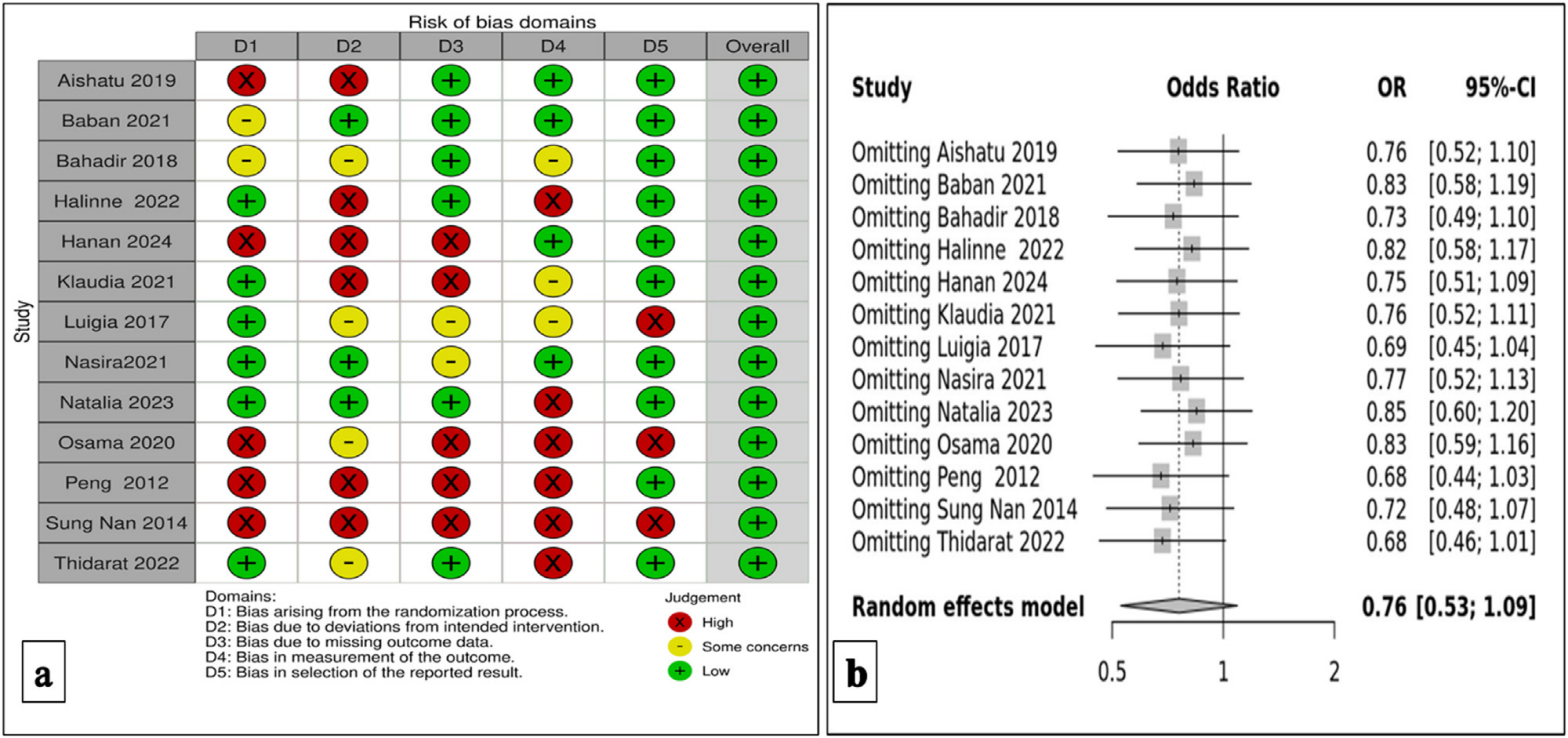
(a): Risk of bias summary and graph for investigating the *TMPRSS6* rs855791 gene polymorphism (b): Sensitivity analysis of rs855791 for both cases and controls.

The Cochrane Rob Tool 2 was utilized to thoroughly measure the methodological quality of the chosen studies, as depicted in Figure 2(a). In this representation, each study is described in a row and each column corresponds to a specific type of bias. The color assigned to each survey indicates the reviewer's evaluation of the bias risk associated with that particular type of analysis. Studies with a low bias risk are represented in green, while those with a high risk of bias are shown in red. Yellow indicates an unclear risk of bias. Overall, the findings suggest a significantly low bias risk for the selected studies, indicating that the research was conducted, executed, and documented to substantially minimize or eliminate potential bias or error.

### Statistical analysis

**Figure 3:**
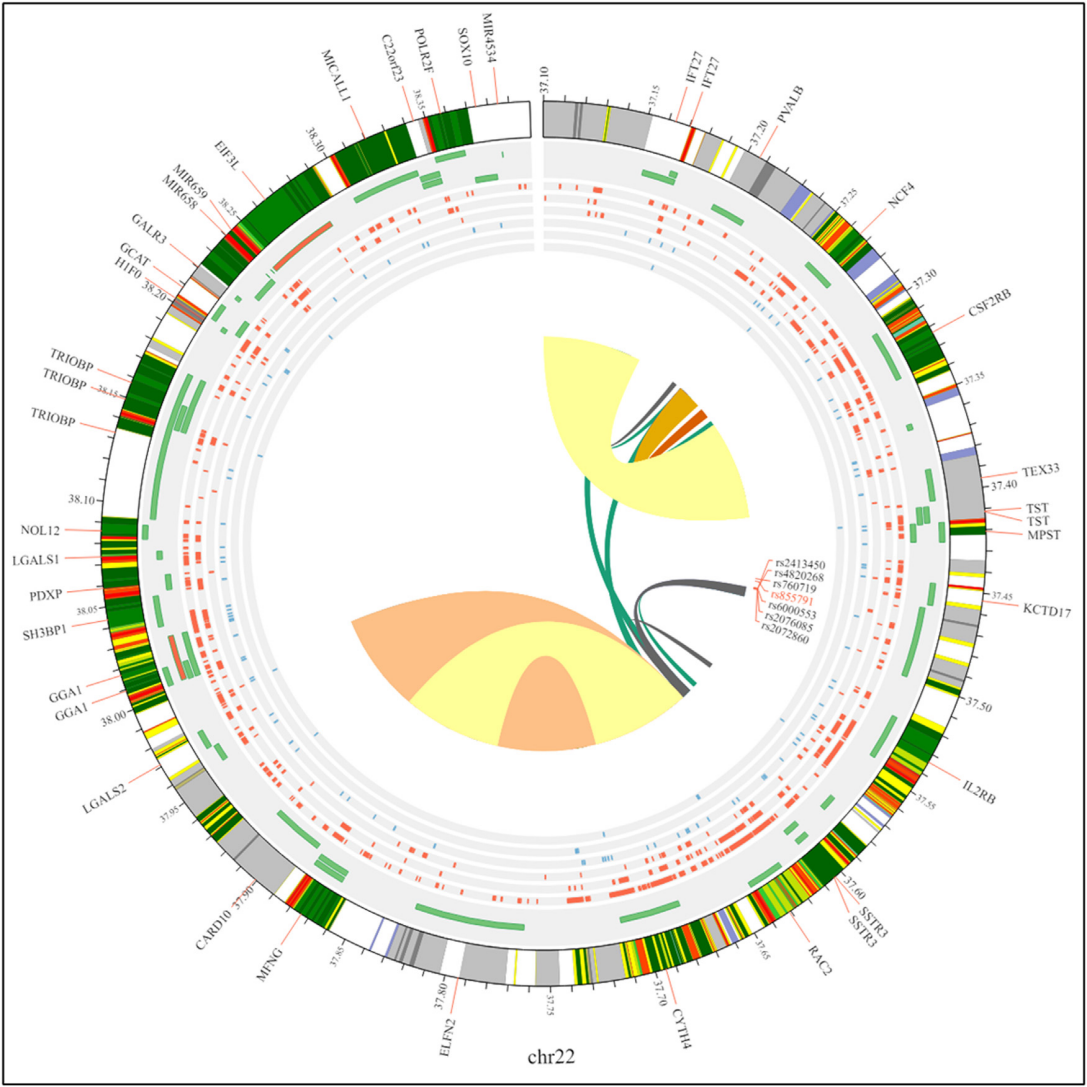
Circos plot showing chromosomal interactions involving the rs855791 single nucleotide polymorphism.

The statistics we analyzed using Review Manager 5.4 software with a statistical consequence threshold of p-value <0.05 for each genetic variation. The chi-square-based Q statistic test was used to examine heterogeneity assumptions across previous research, measured by the I^2^ metric value. In earlier studies, the random-effect model was used to evaluate the odds ratio and 95 % CI, creating a forest plot for ease of evaluation. Additionally, we used a funnel plot to scrutinize potential publication bias within the meta-analysis. The chromosomal interactions with the SNPs are represented by a Circos plot to visualize the complete data using the 3D SNP tool in Figure 3.

## Results

### Study characteristics

Figure 1 thoroughly illustrates the study selection and assessment process, which adheres to rigorous inclusion and exclusion criteria. At the outset, 795 publications were comprehensively gathered and meticulously evaluated to ensure their suitability. The inclusion of thirteen studies in this meta-analysis was based on careful assessment using the HWE score and NOS scale to ensure data accuracy and reliability.^14^, ^15^, ^16^, ^17^, ^18^, ^19^, ^20^, ^21^, ^22^, ^23^, ^24^, ^25^, ^26^ The characteristics of the studies, including their NOS score, are presented, and detailed information concerning genotype distribution, allelic frequency, and HWE/chi-square values for the chosen polymorphisms are provided (Table S1).

### Association between the TMPRSS6 variant with anemia

**Figure 4:**
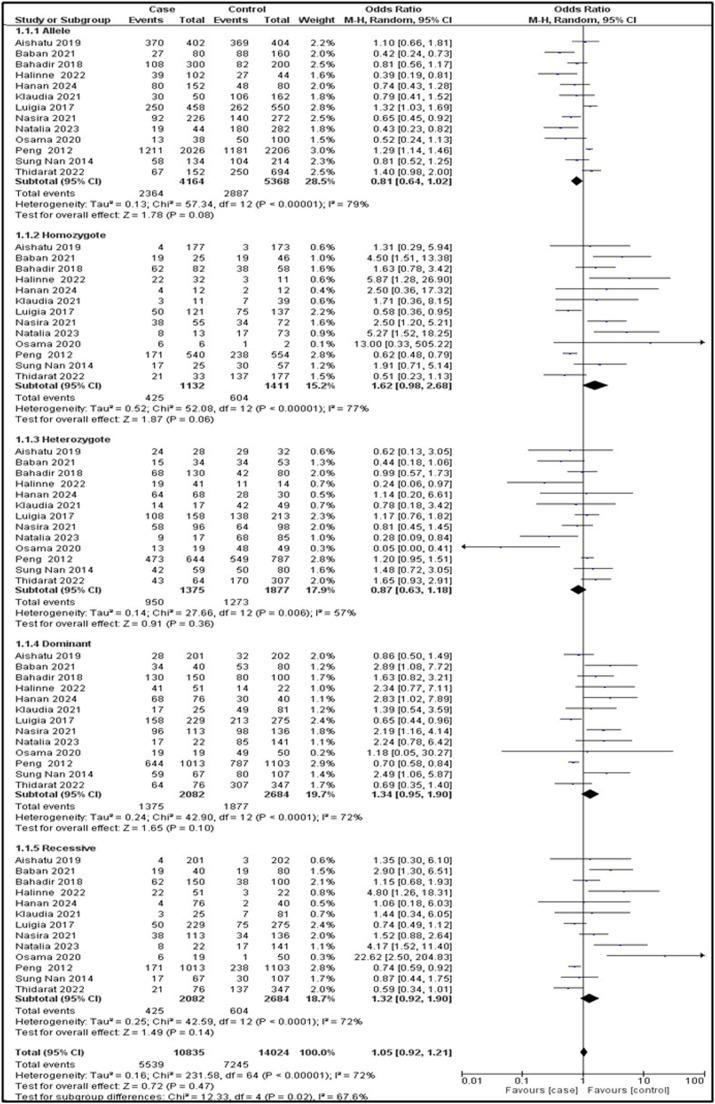
Forest plot showing the association between the *TMPRSS6* rs855791 gene polymorphism and anemia in the genetic model.

Random effects were used because the I^2^ ≥50 % in the allelic model (I^2^ = 79 %; OR = 0.81; 95 % CI: 0.64–1.02; p-value = 0.08), in the homozygous model (I^2^ = 77 %; OR = 1.62; 95 % CI: 0.98–2.68; p-value = 0.06), in the heterozygous model (I^2^ = 57 %; OR = 0.87; 95 % CI: 0.63–1.18; p-value = 0.36), in the dominant model (I^2^ = 72 %; OR = 1.34; 95 % CI: 0.95–1.90; p-value = 0.10), and in the recessive model (I^2^ = 72 %; OR = 1.32; 95 % CI: 0.92–1.90; p-value = 0.14). Overall, none of the five genetic models demonstrated any significant associations. All the data are shown using forest plots. To ascertain the sensitivity of the *TMPRSS6* rs855791 polymorphism, a comprehensive analysis was carried out, encompassing Begg's funnel plot and Egger's test. Figures 4 and S1 reveal no publication bias for the five genetic models in the data.

### An examination of sensitivity analysis

A sensitivity analysis for the *TMPRSS6* gene variation (rs855791) was performed, and the results in Figure 2(b) were similar. We, therefore, conclude that our findings are statistically significant.

## Discussion

Genetic variations in DNA, particularly changes in SNPs, can influence how specific amino acid conversions in a protein affect the functions and activity of a gene.^27^ Genetic variations in the *TMPRSS6* gene impact hematologic parameters and serum iron levels. Additionally, it was discovered that SNPs of the *TMPRSS6* gene were connected to quantitative changes in hematologic markers. Whether the relationship between the *TMPRSS6* gene variations and erythropoiesis relies on iron is unknown.^28^ A study involving female university students in northern Saudi Arabia showed that the *TMPRSS6* polymorphism rs855791 significantly correlated with reduced iron levels. At the same time, rs2111833 did not show such a correlation.^14^ The study provides relevant data concerning the genotype distribution of SNPs in the *TF, TMPRSS6*, and *HFE* genes, along with their potential association with the iron status and blood iron levels of pregnant Filipino women.^29^ Data from Taiwan indicated that reproductive-age women diagnosed with IDA exhibited a reduced frequency of the *TMPRSS6* rs855791 CC genotype compared to women without the condition.^15^ It is essential to acknowledge that IDA is a multifactorial condition influenced by various factors such as nutrition, socioeconomic status, gender, and age. These non-genetic factors are often more prevalent and could overshadow the genetic contributions, making it difficult to detect a direct correlation between *TMPRSS6* polymorphisms and anemia.^30^

Additionally, this study did not differentiate between IDA and iron refractory-iron deficiency anemia (IRIDA), a specific subtype of anemia that is more likely to be influenced by genetic factors such as *TMPRSS6* polymorphisms.^31^ IRIDA, characterized by a poor response to oral iron therapy, has been directly linked to mutations in the *TMPRSS6* gene, with rs855791 being one of the polymorphisms of interest. Studies have shown that individuals with IRIDA are more likely to carry specific *TMPRSS6* variants, which disrupt iron regulation and lead to persistent anemia.^32^

This meta-analysis, which consisted of 2082 cases and 2684 controls, aimed to establish a correlation between anemia and *TMPRSS6* gene polymorphisms with a specific SNP. We tabulated the results using overall OR, a 95 % CI, and p-value. This study unequivocally found no evidence of a relationship between anemia and *TMPRSS6* (rs855791) in all genetic models. Therefore, our findings strongly suggest no potential link between the *TMPRSS6* rs855791 gene polymorphism and anemia. This study provides important insights into the lack of association between anemia and variations in the *TMPRSS6* gene. The results of this study differ from previous studies due to a larger sample size, diverse population coverage, and more rigorous methodologies, including comprehensive genetic model assessments and publication bias evaluations. These factors may have revealed a lack of association that smaller, less robust studies did not detect. Further research is needed to understand how variations in the *TMPRSS6* gene are connected to anemia. Additional studies with more diverse populations are required to explore other *TMPRSS6* polymorphisms, gene-gene interactions, and iron metabolism pathways. Longitudinal studies and functional assays could also provide deeper insights into the gene's role in anemia. Delving deeper could uncover vital information for better diagnostics and treatments. This study is a significant milestone in improving healthcare for individuals with IDA, as it enhances our understanding of this relationship.

## Conclusion

This comprehensive meta-analysis examined the association between the *TMPRSS6* gene polymorphism (rs855791) and anemia, incorporating data from 13 studies comprising over 4700 participants. Contrary to previous hypotheses, our findings revealed no significant correlation between the *TMPRSS6* rs855791 gene variation and anemia across various genetic models. These results suggest that the *TMPRSS6* rs855791 polymorphism may not play a substantial part in predisposing individuals to anemia. However, given the multifactorial nature of anemia and the complexity of genetic influences, further investigation is warranted to elucidate the precise mechanisms underlying anemia development and identify additional genetic factors. Such insights are crucial for refining diagnostic methods and developing targeted therapeutic interventions, ultimately improving clinical management and outcomes for individuals affected by anemia.

## Author contributions

JM wrote the contents, edited the figures and tables of this manuscript. RV designed the study, edited the contents of this manuscript, and approved the manuscript for submission. All authors read and approved the final manuscript.

## Conflicts of interest

The authors declare no conflict of interest to report.

## Appendix Supplementary materials

Image, application 1
