# Supplementary material for: Lack of association between the TMPRSS6 gene polymorphism (rs855791) and anemia: a comprehensive meta-analysis
Source: Hematol Transfus Cell Ther. 2025 Mar 12;47(2):103737. doi: 10.1016/j.htct.2025.103737 (PMC11954102; doi:10.1016/j.htct.2025.103737)
Supplement: Supplementary file 1 [file mmc1.docx]

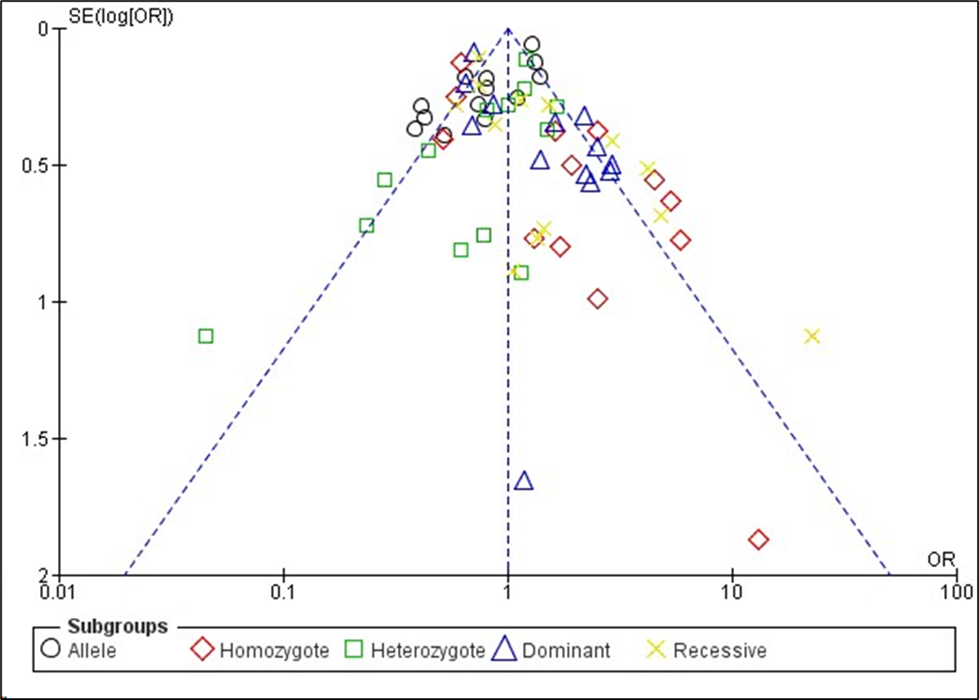


**Figure S1:** Publication bias in the association between *TMPRSS6* gene polymorphism (rs855791) and anemia in genetic models

**Table S1:** Association studies of *TMPRSS6* gene polymorphism with anemia

| **Author &Year** | **Genotypic Frequency** | | | | | | **Allele Frequency** | | | | **Sample size** | | **Ethnicity** | **HWE** | **NOS** | **Reference** |
| --- | --- | --- | --- | --- | --- | --- | --- | --- | --- | --- | --- | --- | --- | --- | --- | --- |
|  | **Case** | | | **Control** | | | **Case** | | **Control** | |  |  |  |  |  |  |
|  | **CC** | **CT** | **TT** | **CC** | **CT** | **TT** | **C** | **T** | **C** | **T** | **Case** | **Control** |  |  |  |  |
| **Aishatu et al., 2019** | 173 | 24 | 4 | 170 | 29 | 3 | 370 | 32 | 369 | 35 | 201 | 202 | South Africa | 0.187 | 6 | 19 |
| **Baban et al., 2021** | 6 | 15 | 19 | 27 | 34 | 19 | 27 | 53 | 88 | 72 | 40 | 80 | Iran | 0.205 | 8 | 20 |
| **Bahadir et al., 2018** | 20 | 68 | 62 | 20 | 42 | 38 | 108 | 192 | 82 | 118 | 150 | 100 | Turkey | 0.187 | 6 | 21 |
| **Halinne et al., 2022** | 10 | 19 | 22 | 8 | 11 | 3 | 39 | 63 | 27 | 17 | 51 | 22 | Sri Lanka | 0.798 | 7 | 22 |
| **Hanan et al., 2024** | 8 | 64 | 4 | 10 | 28 | 2 | 80 | 72 | 48 | 32 | 76 | 40 | Egypt | 0.003 | 8 | 23 |
| **Klaudia et al., 2021** | 8 | 14 | 3 | 32 | 42 | 7 | 30 | 20 | 106 | 56 | 25 | 81 | Poland | 0.188 | 7 | 24 |
| **Luigia et al., 2017** | 71 | 108 | 50 | 62 | 138 | 75 | 250 | 208 | 262 | 288 | 229 | 275 | Italy | 0.922 | 7 | 25 |
| **Nasira et al., 2021** | 17 | 58 | 38 | 38 | 64 | 34 | 92 | 134 | 140 | 132 | 113 | 136 | Pakistan | 0.498 | 6 | 26 |
| **Natalia et al., 2023** | 5 | 9 | 8 | 56 | 68 | 17 | 19 | 25 | 180 | 102 | 22 | 141 | Brazil | 0.597 | 7 | 27 |
| **Osama et al., 2020** | 0 | 13 | 6 | 1 | 48 | 1 | 13 | 25 | 50 | 50 | 19 | 50 | Saudi Arabia | 0 | 8 | 16 |
| **Peng et al., 2012** | 369 | 473 | 171 | 316 | 549 | 238 | 1211 | 815 | 1181 | 1025 | 1013 | 1103 | China | 0.987 | 6 | 28 |
| **Sung Nan et al., 2014** | 8 | 42 | 17 | 27 | 50 | 30 | 58 | 76 | 104 | 110 | 67 | 107 | Taiwan | 0.503 | 7 | 18 |
| **Thidarat et al., 2022** | 12 | 43 | 21 | 40 | 170 | 137 | 67 | 85 | 250 | 444 | 76 | 347 | Thailand | 0.241 | 6 | 29 |

HWE: Hardy-Weinberg equilibrium; NOS: Newcastle-Ottawa Scale
